# The Emaciation Disease: The Possibility of Non-Pathogenic Occurrence in Olive Flounder (*Paralichthys olivaceus*)

**DOI:** 10.3390/ani14223176

**Published:** 2024-11-06

**Authors:** Gyoungsik Kang, Won-Sik Woo, Bong-Jo Kang, Woon-Chul Kang, Chan-Il Park

**Affiliations:** 1 Department of Aquatic Life Medicine, College of Marine Science, Gyeongsang National University, Tongyeong 53064, Gyeongsangnam-do, Republic of Korea; sik27290@gnu.ac.kr; 2 Department of Marine Biology and Aquaculture, College of Marine Science, Gyeongsang National University, Tongyeong 53064, Gyeongsangnam-do, Republic of Korea; dnstory@hanmail.net (W.-S.W.);; 3 Ocean and Fisheries Research Institute, Seogipo 62629, Jeju Special Self-Governing Province, Republic of Korea

**Keywords:** emaciation disease, non-pathogenic disease, *Enteromyxum leei*, *Parvicapsula anisocaudata*

## Abstract

Emaciation disease has directly affected the production of olive flounder—a major aquaculture species in Korea—since the 2000s. Various myxosporeans have been reported as its causative agents. In this study, we investigated the recent resurgence of emaciation on Jeju Island, considering not only myxosporean infections but also potential environmental factors. Unlike previous studies that have primarily attributed the causes of emaciation disease to infections by myxosporeans, this research aimed to determine whether pathogen infection is indeed the sole cause of emaciation disease through molecular and histopathological analyses. Molecular biological and histopathological analyses of aquaculture and laboratory samples revealed that gross pathology lesions of emaciation can occur in the absence of pathogens, as well as in environments with myxosporean infections. These findings suggest that emaciation disease can be exacerbated by certain environmental factors in addition to myxosporeans. Future research should focus on detailed experimental designs to identify the specific environmental conditions contributing to the severity of this disease.

## Simple Summary

Emaciation disease has significantly impacted olive flounder production in Korea since the 2000s, with various myxosporeans identified as causative agents. This study on Jeju Island examined both myxosporean infections and environmental factors, finding that emaciation lesions can occur without pathogens. The results indicate that environmental factors, along with myxosporeans, can worsen the disease. Future research should aim to pinpoint specific environmental conditions that contribute to its severity.

## 1. Introduction

The first report of emaciation in aquaculture species was in gilthead sea bream (*Sparus aurata*), and since then, it has been continuously documented in various species, including tiger puffer (*Takifugu rubripes*), turbot (*Scophthalmus maximus*), olive flounder (*Paralichthys olivaceus*), and starry flounder (*Platichthys stellatus*) [1,2,3,4]. In particular, it has caused significant issues in olive flounder (*P. olivaceus*), a major aquaculture species in South Korea, which has been continuously documented since its first report in 2007, and accounts for 50% of finfish aquaculture in the country, highlighting its importance within the industry [3,5,6,7,8,9].

Myxosporeans such as *Enteromyxum leei*, *E. scophthalmi*, *Parvicapsula anisocaudata*, and *Sphaerospora fugu* have been suggested as causative agents of emaciation disease, which is characterized by significant lesions of emaciation in affected individuals [1,2,3,9,10,11]. Particularly on Jeju Island, where emaciation disease is a significant problem in South Korea, *E. leei* and *P. anisocaudata* have been identified as the primary causative agents [3,5,6,7,9,12]. Moreover, these pathogens have recently spread to another major aquaculture species in Korea, starry flounder (*Platichthys stellatus*), causing significant issues [4].

Jeju Island has consistently reported damage due to emaciation disease caused by *E. leei* and *P. anisocaudata* [8,9]. However, with changes in aquaculture practices, the impact of emaciation disease has recently spread. Historically, aquaculture on Jeju Island employed a method combining groundwater and seawater. Recently, structural changes in aquaculture farms have eliminated the mechanical system for dropping groundwater, which has coincided with an increase in mortality due to emaciation disease. This study hypothesized that emaciation disease can occur solely due to gas concentrations similar to those found in aquaculture, regardless of pathogen infection status, and examined individuals exhibiting gross pathology lesions of emaciation. Furthermore, unlike previous studies that have primarily attributed the causes of emaciation disease to infections by myxosporeans, this research aimed to determine whether pathogen infection is indeed the sole cause of emaciation disease through molecular and histopathological analyses.

## 2. Materials and Methods

### 2.1. Fish

Gross pathology of emaciation disease was analyzed in a total of 30 olive flounders, divided into three groups: 20 fish (10 from each of two aquaculture farms) from Jeju Island exhibiting natural gross pathology of emaciation disease and 10 fish from a laboratory setting under identical environmental conditions but without pathogen infection. The laboratory samples were collected from the aquaculture farm only after confirming the absence of two parasites associated with emaciation disease, *E. leei* and *P. anisocaudata*. The samples used for analysis were acclimated in land-based tanks with aeration, utilizing underground seawater with a temperature of approximately 18.8 ± 0.1 °C, dissolved oxygen levels of approximately 3.8 ± 0.1 mg/L, and an oxygen saturation of 49%.

### 2.2. Histopathology

After excising the brains, eyes, digestive tracts (stomachs and intestines), gills, hearts, anterior and posterior kidneys, livers, and spleens from olive flounder (*P. olivaceus*), these tissues were initially fixed in 70% ethanol for 24 h and subsequently re-fixed in 10% neutral formalin for an equivalent duration. The tissues then underwent dehydration in a graded series of ethanol (70% to 100%) and clearing with xylene using an automated tissue processor, followed by paraffin embedding. Sections were cut at a thickness of 4 μm and stained with hematoxylin and eosin (H&E) for examination under a light microscope.

### 2.3. Molecular Biology

Genomic DNA was extracted individually from all excised organs of the olive flounder (*P. olivaceus*) utilizing the AccuPrep^®^ Genomic DNA Extraction Kit (Bioneer, Daejeon, Republic of Korea) in accordance with the manufacturer’s instructions. The extracted genomic DNA samples were stored at −80 °C until further analysis. For diagnosis of the pathogen causing emaciation disease, PCR was conducted using specific primers (Table 1). The PCR mixture contained 10 μL of Exprime Taq Premix (GeNet Bio, Nonsan, Republic of Korea), 7 μL of distilled water, 1 μL of genomic DNA, and 1 μL each of forward and reverse primers.

Prior to sequence analysis, the PCR amplicons were purified using the QIAquick^®^ Gel Extraction Kit (Qiagen, Hilden, Germany) as per the manufacturer’s protocol. The purified PCR products were then cloned into the pGEM^®^-T Easy Vector (Promega, Madison, WI, USA) and transformed into *Escherichia coli* JM109 competent cells following the standard procedures. After sufficient propagation, plasmid DNA was extracted using the Hybrid-Q™ Plasmid Rapidprep Kit (GeneAll^®^, Seoul, Republic of Korea) and sequenced with the universal M13 primer set. Nucleotide sequence analysis for the identification of the nervous necrosis virus was performed using the Basic Local Alignment Search Tool (BLAST) algorithm provided by the National Center for Biotechnology Information (https://blast.ncbi.nlm.nih.gov/blast accessed on 1 August 2024).

## 3. Results

### 3.1. The Condition Factor and Gross Pathology Lesions

The aquaculture samples were divided into two groups: a small group with a relatively short duration of aquaculture and a large group with a longer duration of aquaculture. In the laboratory, olive flounders of a similar size to those in the large group were used.

The condition factor was calculated using total length and body mass, and the relative condition factor (rCF) was also derived following the methodology of Shin et al. (2018) [3] (Table 2). The results indicated that the severity of emaciation disease was highest in the aquaculture group (small), followed by the laboratory group, and then the aquaculture group (large) (Table 2). Furthermore, according to the criteria presented in Table 3, all samples were confirmed to have severe infections at a level detectable for parasites.

**Figure 1:**
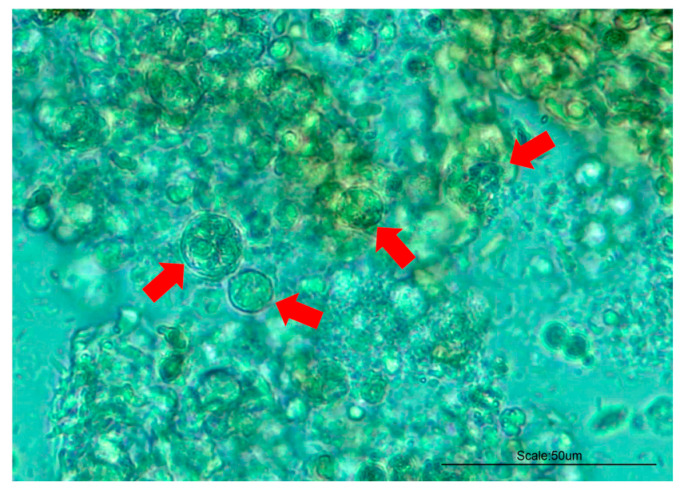
Optical microscopic observation of the myxosporeans isolated from the intestine (indicated by red arrows).

**Figure 2:**
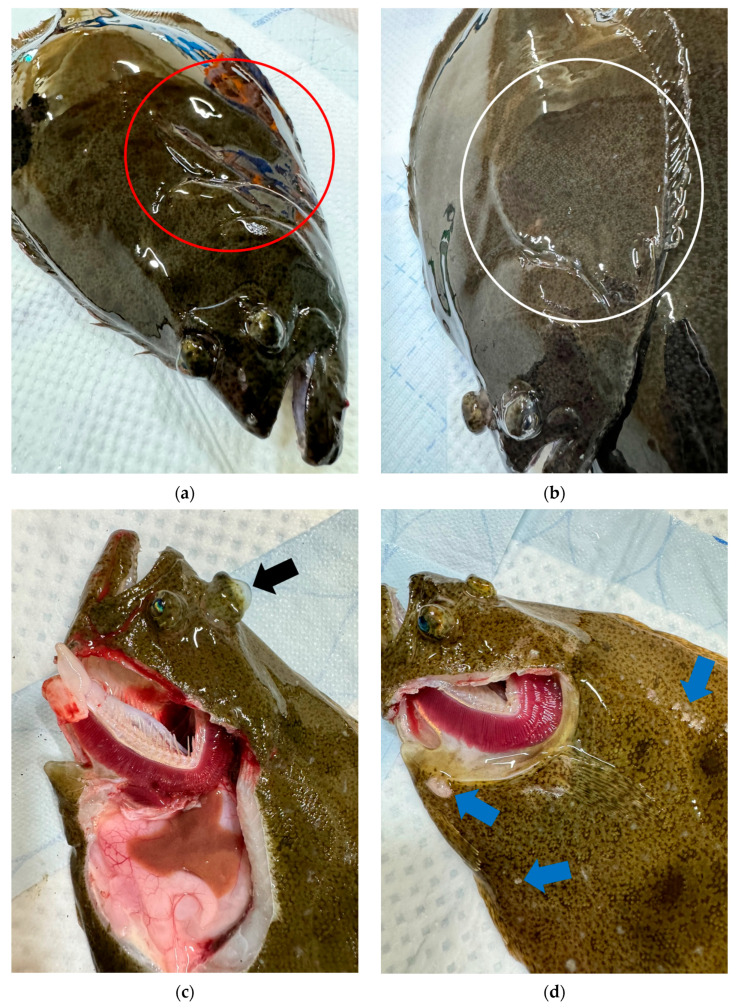
Gross pathology of olive flounders. (**a**) represents the aquaculture group, while (**b**–**d**) represent the laboratory group. Red and white ellipses indicate emaciation lesions, a black arrow indicate emboli in the eyes, and blue arrows indicate vesicular lesions.

After scraping the intestines of the analyzed olive flounders with a scraper and observing them under an optical microscope, myxosporeans were detected in all the aquaculture samples (Figure 1). Additionally, the observed gross pathology included lesions of emaciation (Figure 2a,b), emboli in the eyes (Figure 2c), and vesicular lesions on the skin (Figure 2d). Particularly, the formation of abdominal invagination is a significant gross pathology lesion of emaciation, serving as a key indicator to reference when clinically suspecting the occurrence of emaciation disease in aquaculture farms. The frequency and severity of these conditions did not show notable variation across different groups or sizes, and they were observed at a similar frequency in all groups.

### 3.2. Histopathological Results

**Figure 3:**
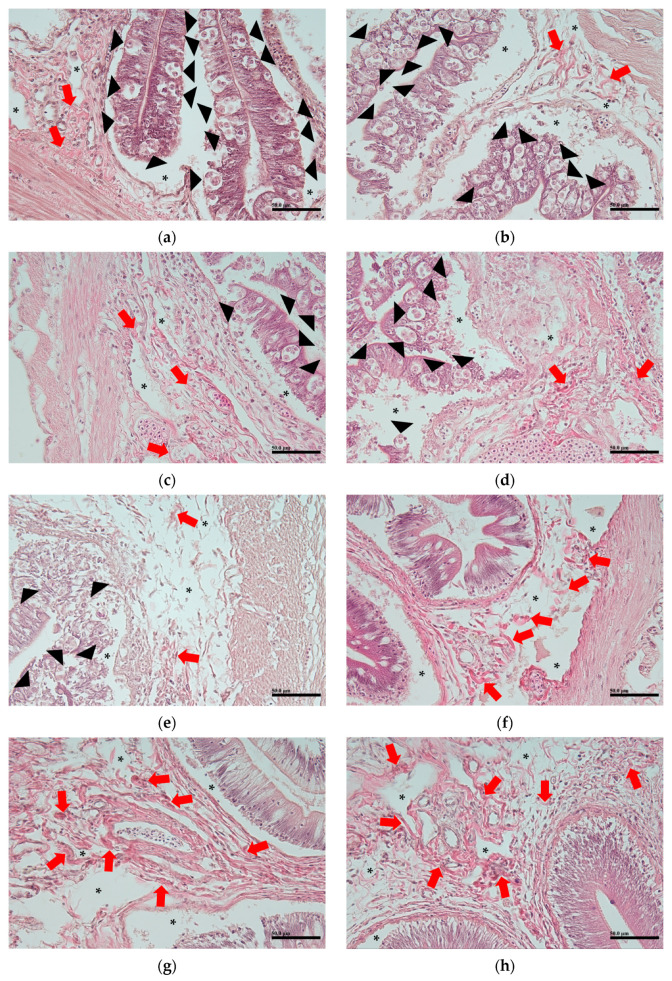
Histopathology of the intestine. (**a**,**b**) represent the smaller aquaculture group, (**c**–**e**) represent the large aquaculture group, and (**f**–**h**) represent the laboratory group. Black arrowheads indicate myxosporeans, black asterisks denote ballooned submucosa, and red arrows indicate infiltration of fibrous tissue. Scale bar = 50 μm.

Histopathological observations revealed that myxozoans, presumed to be the *E. leei* or *Parvicapsula* sp., were present in the intestines of all the aquaculture samples (Figure 3a–e). In contrast, the laboratory samples did not show any presence of myxozoans or lesions indicative of the *E. leei* or *Parvicapsula* sp. (Figure 3f–h).

Figure 3a shows submucosa that closely resembles normal tissue. In contrast, the other figures exhibit ballooned submucosa, which is presumed to be caused by environmental conditions. Notably, the samples from the laboratory group exhibit ballooning of the submucosa, along with prominent infiltration of fibrous tissue (Figure 3f–h).

**Figure 4:**
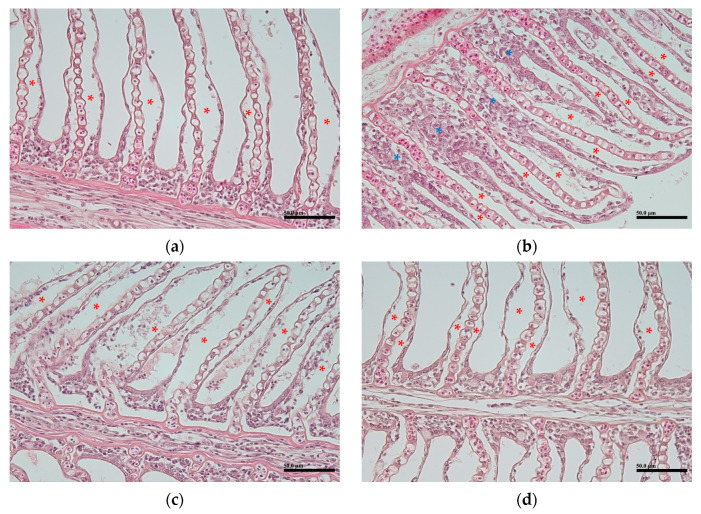
Histopathological observations of the gills. (**a**) shows mild epithelial cell lifting in the large aquaculture group, (**b**) shows moderate epithelial cell lifting in the small aquaculture group, (**c**) shows severe epithelial cell lifting in the large aquaculture group, and (**d**) shows mild epithelial cell lifting in the laboratory group. Red asterisks indicate epithelial cell lifting, and blue asterisks indicate infiltration of inflammatory cells. Scale bar = 50 μm.

Another significant histopathological lesion observed was the lifting of the gill epithelial cells, with no differences noted between sizes or groups (Figure 4). In addition, infiltration of the inflammatory cells was observed in some individuals, but no significant differences were noted (Figure 4b).

### 3.3. Definition Diagnosis

Molecular biological analysis revealed that *E. leei* and *P. anisocaudata* were detected in all the aquaculture samples, regardless of size. In contrast, no myxosporeans were detected in the laboratory samples. Additionally, the biopsy results also supported the presence of myxosporean infection in the aquaculture samples (Table 4). However, while all the laboratory samples exhibited clinically confirmed emaciation lesions, no myxosporean infection was detected (Table 4).

## 4. Discussion

Emaciation disease has predominantly been studied as an infectious disease caused by pathogens [1,2,3,4,9,10,11]. However, in this study, we identified that emaciation disease can occur in the absence of myxozoan infection. Notably, the gross pathology of emaciation disease was consistently observed in samples maintained under identical environmental conditions in the laboratory, even in the absence of pathogens. This finding supports the possibility that the recent increase in emaciation disease cases on Jeju Island may be attributed not only to an increase in pathogens but also to environmental factors in aquaculture farms. Gross pathology of emaciation disease has been observed due to environmental conditions, and unlike in a laboratory setting, pathogen control has been challenging in aquaculture facilities. Consequently, it was presumed that the mortality associated with emaciation disease may have worsened over the long term, indicating a persistent impact.

Histopathological analysis of the intestine revealed ballooned submucosa lesions, likely caused by environmental conditions, which may have impeded the proper transfer of nutrients absorbed by the intestinal villi of the olive flounder into the body. Additionally, these lesions were observed in both the aquaculture group (with pathogens) and the laboratory group (without pathogens). Since all these samples exhibited the gross pathology of emaciation disease, this suggests that such gross pathology can arise from environmental factors in addition to pathogen infection.

Although myxosporean infections were detected in the laboratory samples, one might question whether they were inadvertently undetected through PCR or histopathology; however, the probability of this is extremely low. Furthermore, the laboratory conditions were designed to mirror those of the aquaculture, excluding pathogen control measures (such as filtration, disinfection, and UV treatment), making it reasonable to conclude that the laboratory samples were free of pathogen infection. However, as not all seawater environmental indicators were measured, future research should establish methods for experimental infections to compare the pathogen differences between aquaculture and laboratory samples, thereby addressing this limitation.

While diagnosis in infected cases is typically suggested through the condition factor, the relative condition factor, and parasite detection via PCR [3,9,16], the possibility of non-pathogenic diseases also exists. Additionally, even if PCR bands are detected, their number may be extremely low, making it advisable to complement molecular diagnosis with histopathological examination. Furthermore, a qPCR diagnostic method has been developed [16]. Utilizing such diagnostic methods, it is essential to conduct systematic and detailed experiments to determine whether the absence of pathological symptoms is due to low-level infections or the release of parasites post-infection or whether emaciation occurs independently of infection solely due to gas regulation.

## 5. Conclusions

Emaciation disease has significantly impacted olive flounder production in Korea since the 2000s, traditionally attributed to myxosporean infections. However, this study on Jeju Island revealed that emaciation lesions can also arise due to environmental factors, independent of the presence of pathogens. Histopathological analyses indicated that environmental-condition-induced ballooned submucosa lesions may hinder nutrient absorption, contributing to emaciation. Furthermore, molecular and histopathological examinations found no infections in the laboratory samples, suggesting non-pathogenic causes. These findings emphasize the need for detailed studies to identify specific environmental conditions affecting disease severity and to utilize comprehensive diagnostic methods, including qPCR, for accurate disease management. However, to address the limitations of this study, additional research is necessary to investigate which environmental factors may contribute to emaciation under laboratory conditions.

## Figures and Tables

**Table 1:** Primers and PCR conditions used in this study.

Target	Primers	Sequences (5′—3′)	Condition *	Size (bp)	References
*Enteromyxum leei*	EL-F	GATGAAACTGCGAAGCGCTC	95 °C for 3 min, (95 °C for 30 s, 55 °C for 30 s, 72 °C for 30 s) ×35 cycles, 72 °C for 7 min	1468	[13]
	EL-R	CACAAGTTGATGACTTGCGC			
*Parvicapsula* sp. (*P. anisocaudata* *)	EM-F	CAACCGCAATGTGTTTACTC		812	[7]
	EM-R	CCAAACAACCTGCCACAATG			

* PCR was performed under the same conditions for both primer sets.

**Table 2:** Results of biomass measurements and the calculation of the condition factor and the relative condition factor.

Group	Body Mass (g)	Total Length (cm)	Condition Factor	rCF *
Aquaculture (small)	152.5 ± 34.5	27.0 ± 1.3	0.77 ± 0.08	68%
Aquaculture (large)	660.0 ± 148.5	40.3 ± 2.3	1.00 ± 0.08	81%
Laboratory	434.0 ± 138.7	36.5 ± 2.8	0.87 ± 0.13	72%

* Relative condition factor.

**Table 3:** Interpretation of the criteria for condition factor and relative condition factor.

**Condition Factor [14]**	**Mean**
Over 1.6	Excellent
1.4 to 1.6	Good
1.0 to 1.2	Fair
0.8 to 1.0	Poor
Under 0.8	Extremely poor
**Relative Condition Factor** [3,15]	**Mean**
Over 100%	Normal
90 to 100%	Condition that may clinically exhibit lesions of emaciation disease
80 to 90%	The detection rate of the myxosporean is moderate, but *E. leei* is identified to be in a relatively less mature stage
70 to 80%	The detection rate of the myxosporean is moderate, and *E. leei* is identified to be in a relatively mature stage
Under 70%	The detection rate of the myxosporean is severe, and severe emaciation is observable to the naked eye

**Table 4:** Results of myxosporean analysis and gross pathology observations of emaciation disease.

Group	Biopsy	Molecular Biology		Gross Pathology	Histopathology *
Aquaculture (small)	10/10 (100%)	*E. leei*	10/10 (100%)	10/10 (100%)	10/10 (100%)
		*P. anisocaudata*	10/10 (100%)		
Aquaculture (large)	10/10 (100%)	*E. leei*	10/10 (100%)	10/10 (100%)	10/10 (100%)
		*P. anisocaudata*	10/10 (100%)		
Laboratory	0/10 (0%)	*E. leei*	0/10 (0%)	10/10 (100%)	0/10 (0%)
		*P. anisocaudata*	0/10 (0%)		

* Accurate species identification is challenging through histopathological analysis.

## Data Availability

The datasets used and/or analyzed during the current study are available from the corresponding author upon reasonable request.
